# Rheumatic heart disease in pregnancy and neonatal outcomes: A systematic review and meta-analysis

**DOI:** 10.1371/journal.pone.0253581

**Published:** 2021-06-29

**Authors:** Joshua Liaw, Betrice Walker, Leanne Hall, Susan Gorton, Andrew V. White, Clare Heal

**Affiliations:** 1 College of Medicine and Dentistry, James Cook University, Mackay, Queensland, Australia; 2 College of Medicine and Dentistry, James Cook University, Townsville, Queensland, Australia; University of Western Australia, AUSTRALIA

## Abstract

**Purpose:**

Associations between rheumatic heart disease (RHD) in pregnancy and fetal outcomes are relatively unknown. This study aimed to review rates and predictors of major adverse fetal outcomes of RHD in pregnancy.

**Methods:**

Medline (Ovid), Pubmed, EMcare, Scopus, CINAHL, Informit, and WHOICTRP databases were searched for studies that reported rates of adverse perinatal events in women with RHD during pregnancy. Outcomes included preterm birth, intra-uterine growth restriction (IUGR), low-birth weight (LBW), perinatal death and percutaneous balloon mitral valvuloplasty intervention. Meta-analysis of fetal events by the New-York Heart Association (NYHA) heart failure classification, and the Mitral-valve Area (MVA) severity score was performed with unadjusted random effects models and heterogeneity of risk ratios (RR) was assessed with the I^2^ statistic. Quality of evidence was evaluated using the GRADE approach. The study was registered in PROSPERO (CRD42020161529).

**Findings:**

The search identified 5949 non-duplicate records of which 136 full-text articles were assessed for eligibility and 22 studies included, 11 studies were eligible for meta-analyses. In 3928 pregnancies, high rates of preterm birth (9.35%-42.97%), LBW (12.98%-39.70%), IUGR (6.76%-22.40%) and perinatal death (0.00%-9.41%) were reported. NYHA III/IV pre-pregnancy was associated with higher rates of preterm birth (5 studies, RR 2.86, 95%CI 1.54–5.33), and perinatal death (6 studies, RR 3.23, 1.92–5.44). Moderate /severe mitral stenosis (MS) was associated with higher rates of preterm birth (3 studies, RR 2.05, 95%CI 1.02–4.11) and IUGR (3 studies, RR 2.46, 95%CI 1.02–5.95).

**Interpretation:**

RHD during pregnancy is associated with adverse fetal outcomes. Maternal NYHA III/IV and moderate/severe MS in particular may predict poor prognosis.

## Introduction

The global prevalence of rheumatic heart disease (RHD) is 1%, and is twice as common in women than men, particularly in women of childbearing age [1, 2]. This figure is likely underestimated in developing countries [2]. RHD accounts for approximately 30% of cardiac disease in pregnancy in developed countries, and 90% of cardiac disease in non-industrialized regions [3, 4].

Normal hemodynamic changes of pregnancy impose an additional 30–50% cardiac load. This is well tolerated by a normal heart but can result in morbidity and mortality in women with pre-existing RHD [5–7]. Mitral stenosis (MS) is especially sensitive to cardiac insufficiency in pregnancy [8, 9]. The placental-fetal heart circulation is likely affected [10], and hemodynamic insufficiency poses a risk to the developing fetus. Complications such as intra-uterine growth restriction (IUGR) and prematurity may have lasting developmental effects into childhood and beyond [11].

The New York Heart Association (NYHA) functional classification of heart failure is used worldwide, with four categories (I-IV) based on limitations during physical activity; Class I–no limit, to Class IV- symptoms at rest [12]. In addition, MS severity can be graded using echocardiography based on mitral-valve area (MVA) into mild (>1.5cm^2^), moderate (1.0–1.5cm^2^) and severe (<1.0cm^2^) [13]. Increasing severity of these indicators (NYHA, MVA) is associated with increased frequency of maternal cardiac complications [9]. In contrast, the association with adverse fetal and neonatal outcomes is often unreported.

The purpose of this study was to review rates of adverse fetal and neonatal outcomes for women with RHD in pregnancy and investigate the association between increasing severity of RHD using the NYHA and MVA scales with fetal outcomes. Additionally, the effects of percutaneous balloon mitral valvuloplasty (PBMV) on fetal events is reported.

## Methods and analysis

This systematic review and meta-analysis is reported in accordance with the PRISMA guidelines [14], and registered with PROSPERO (CRD42020161529) [15].

### Search strategy

An electronic search of Medline (Ovid), Pubmed, EMcare, Scopus, CINAHL, Informit, and WHO ICTRP was performed on 15 July 2020, limited to studies published in English language between 01 January 1990–15 July, 2020.

The complete search strategy (S1 Fig) used combined controlled vocabulary with free-text words related to population, intervention/exposure, and outcome (PICO). Studies were eligible for inclusion if they were conducted at a tertiary centre and reported associations between RHD in pregnancy and one or more pre-specified fetal outcomes. Studies with non-specific pregnancy-related cardiac disease, concordant congenital heart disease, isolated pulmonary or aortic valve involvement were excluded. Randomized controlled trials, intervention studies, cohort studies, case-control studies were eligible for inclusion. Case reports, case series, reviews, and duplicates were excluded.

Titles and abstracts were screened by the primary author (JL) on selection criteria. A second reviewer (BW) screened a sample until agreement reached >0.8 using Cronbach alpha [16]. For all selected articles, the full text were retrieved and evaluated by primary author and independent second reviewer (BW) for eligibility. In case of disagreements, a third reviewer was consulted (CH), and a decision agreed by consensus. Additional studies were identified from a manual search of references of included studies.

### Type of outcome measures

Studies reporting one or more of the following outcomes were included: preterm birth (live delivery before 37 weeks gestation), low birth weight (LBW) (<2500 grams), small for gestational age (SGA) or intra-uterine growth restriction (IUGR) (estimated weight <10% percentile for gestational age), miscarriage (non-viable products of conception <20 weeks gestation) or perinatal death (including stillbirths (fetal demise after 20 weeks) and neonatal deaths (within the first 28 days of life)).

### Data extraction and risk of bias assessment

Data were extracted into custom data collection forms by two independent reviewers (JL, BW). Authors were contacted for further information if required. Information extracted included: authors, setting, location, study design, study period and population characteristics (maternal age, gravida, parity, gestational age, rheumatic valvar lesions, mitral valve area severity, baseline NYHA classifications and mode of delivery).

Two authors (JL and BW) independently scored the risk of bias with a modified Quality in Prognostic Studies (QUIPS) tool [17] (S2 Fig). A risk of bias assessment was based on criteria for study participation, study attribution, prognostic factor measurement, outcome measurement, study confounding and statistical analysis. Each category was classified as low, medium, high or unknown risk of bias. Discrepancies were resolved by consensus or by a third reviewer (LH).

### Statistical analysis

Rates of neonatal outcomes were recorded and compared based on maternal baseline NYHA status and MVA severity at the time of first antenatal visit. The effect of minimal invasive intervention (PBMV) during pregnancy on neonatal outcomes was narratively synthesised. An a-priori decision was made to perform meta-analysis if sufficient data was available. Weight of the studies in the meta-analysis was calculated based on the Mantel-Haenszel test using Revman v5.4 [18]. The random effects model was chosen to account for inter- and intra-study variability [19, 20]. Between-study heterogeneity was assessed using the I^2^ test [21]. Risk ratios (RR) were reported with 95% confidence intervals (CI). Subgroup analysis was conducted based on country and non-PBMV/PBMV cohorts and sensitivity analysis was conducted with exclusion of outliers. Small study bias (including publication bias) was examined using funnel plots and Egger’s test [22] if 10 or more studies were available, with statistical significance set at 10%.

### Evaluating the presented evidence

The Grades of Recommendation, Assessment, Development and Evaluation (GRADE) [23] system was used to evaluate the certainty of evidence across studies regarding clinical significance of NYHA or MVA on fetal outcomes.

## Results

**Fig 1:**
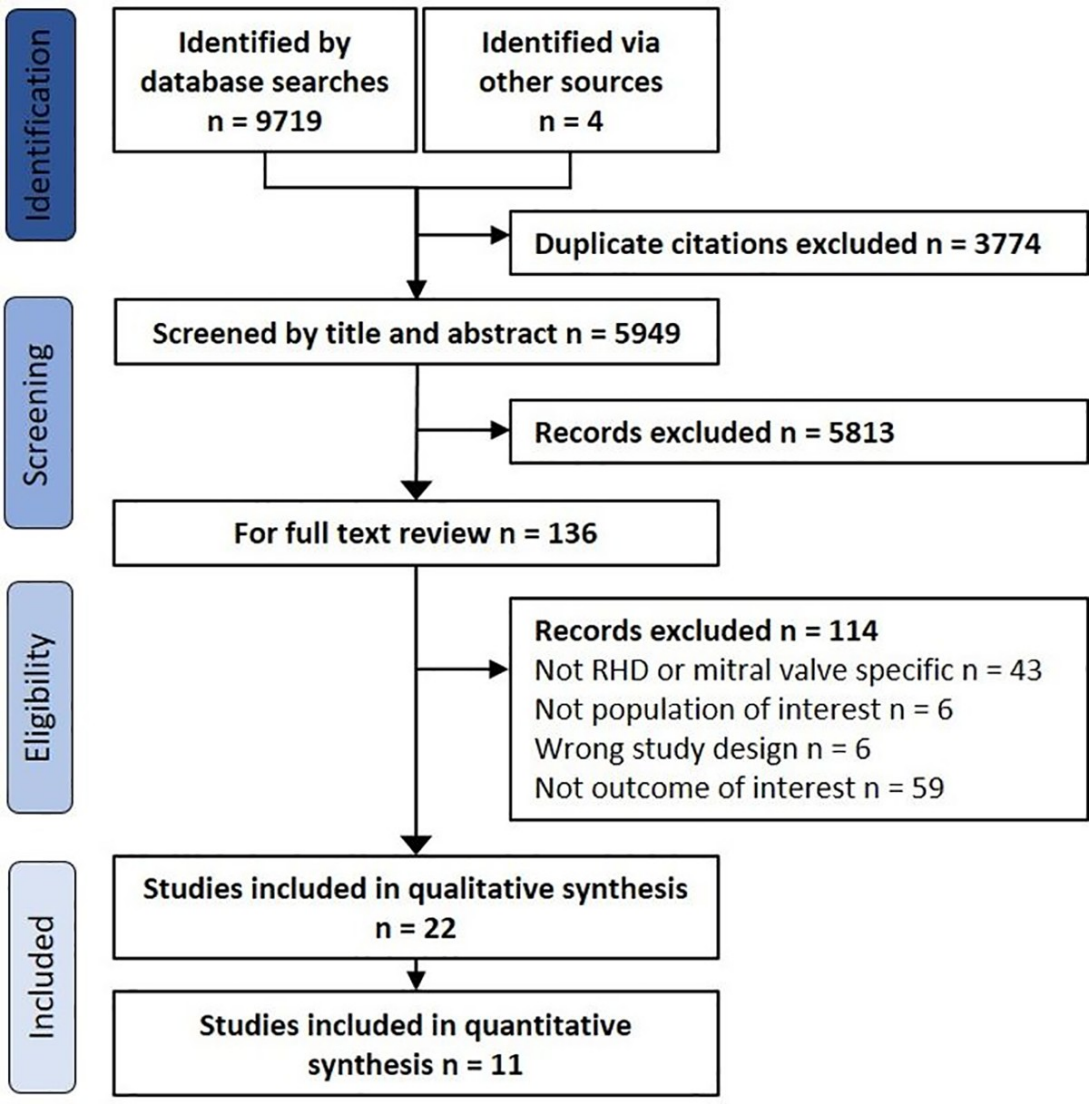
Study flow diagram.

The initial database search identified 9719 papers. A further 4 papers were obtained from other sources. After removal of duplicates (n = 3774) and ineligible studies from title and abstract screening (n = 5813) and full text review (n = 114), 22 studies were included in the review (all cohort study designs) (Fig 1), and 11 in the meta-analysis (Table 1).

**Table 1:** Characteristics of included studies.

Author (year)	Country and setting	Study design	Sample size (n) pregnancies	Population	QUIPS (Risk of bias)	Additional Comments
Bhatla et al. (2003) [27]	India, New Delhi	Retrospective	183	Maternal age– 25.66 +/-3.90	Moderate	Outcomes were for both RHD and congenital heart disease.
				NYHA III/IV– 12.0%		Study time frame not specified.
				Previous surgery– 9.4%		
				8.1% mothers had unspecified respiratory disease		
Suri V, et al. (2019) [36]	India, Chandigarh	Retrospective	309	NYHA III/IV –14.2%	Moderate	Average diagnosis of RHD at 26 weeks GA
						Education low– 14% illiterate, 10.6% primary school
				Previous surgery– 9.38%		
				Severe MS– 36.67%		
				Maternal age– 25.6 +/- 3.9		
Sawhney H, et al. (2002) [33]	India, Chandigarh	Retrospective	500	Maternal age– 25.27 +/- 3.79	Moderate	35 pregnancies lost to follow up.
				NYHA III/IV– 22.6%		Unclear definitions of perinatal death.
				Previous Surgery– 35.6%		
Mane SV, et al. (1993) [26]	India, Mumbai	Retrospective	51	Maternal age–not reported	High	LBW was defined as <2000g.
				NYHA III/IV– 46.0%		No report on antenatal care, GA, co-morbidities.
				Maternal anaemia– 13.7%		Unclear timing of NYHA measurement.
Pandey U (2014) [31]	India, Varanasi	Retrospective	96	Maternal age– 79% between 21–35 years old	Moderate	Timing of NYHA measurement was not specified.
						Unclear definition of preterm birth.
				NYHA III/IV = 4.2%		
				All booked antenatal care by 6-18weeks		
Shuchi J (2013) [29]	India, Kolkata	Retrospective	48	Maternal age– 25 +/- 3.4	Moderate	Did not report patients with previous cardiac surgery.
				NYHA III/IV– 25.0%		
Brezinov OP, et al. (2019) [8]	Israel, Tel Hasomer	Retrospective	31	Maternal age– 30.97 +/- 5.59	Moderate	35 pregnancies were lost to follow up.
				NYHA III/IV– 9.7%		Did not report any comorbidities.
				Previous surgery– 51.61%		
				Severe MS– 22.5%		
Baghel J, et al. (2020) [7]	South India, Puducherry	Retrospective	820	Maternal age– 25.3+/-4.4	Low	26.3% were diagnosed during pregnancy.
				Prior cardiac intervention -22.4%		Main outcomes were to create a predictor score for adverse maternal cardiac events in pregnancy.
				NYHA III/IV– 1.2%		
				33.9% had anaemia		
Nqayana et al. (2008) [38]	South Africa, Durban	Retrospective	77	Maternal age– 21–40	Low	Definition for LBW was <2kg.
				28% patients had MS		
				NYHA III/IV– 31.2%		
				Previous surgery– 60.6%		
				HIV +ve in 32.6%		
Desai DK, et al. (2000) [28]	South Africa, Durban	Prospective	128	Maternal age– 27.00	Moderate	42% new diagnosis in pregnancy.
				NYHA–NA		Did not specify exact years of study.
				Severe MS– 29.0%		
				Anaemia– 25%		
				Warfarin and heparin– 13%		
Sharma P, (2017) [34]	Nepal, Kathmandu	Prospective	85	Maternal age– 27.34	Moderate	Excluded mitral valve repair/replacement, MVA >1.5cm^2^ and patients that did not receive any antenatal care.
				NYHA III/IV– 20.0%		
				Previous operated–Not reported		
						NYHA was measured throughout pregnancy and unclear which was reported in final report.
				60% primipara		
Chhetri S, (2014) [25]	Nepal, Eastern Nepal	Prospective	45	Maternal age– 25 +/- 5	High	<90% presented for first time at labour
				NYHA III/IV– 33.3%		No inclusion or exclusion criteria, and no record on past surgery, anticoagulation or comorbidities.
				Severe MS– 8.9%		
				38% had pulmonary hypertension		Unclear when NYHA was assessed.
Van Hagen et al. (2019) [9]	ROPAC*	Prospective	390 (218 with MS +/- MR)	NYHA >1–43.6% (of 390)	Low	Over 60 countries are involved in this registry.
				Severe MS– 24.77% (of 218)		
Barbosa PJB et al. (2000) [24]	Brazil, Salvador	Retrospective	45	Maternal age– 28.8 +/- 4.6	High	NYHA was collected on follow up post-pregnancy for 6 patients.
				NYHA III/IV– 86.6%		
				Severe MS– 42.2%		Did record of past surgery, anticoagulants or RHD specific lesions.
Sartain JB, et al. (2012) [41]	Australia, Cairns	Retrospective	74	Maternal age–not stated.	Low	Only 74/94 infant data was available (<80%). No reason for loss of follow up given.
				*(High cardiac risk score)– 5.55%		
				Previous operated– 8.10%		
Ongzalima C, et al. (2019) [39]	Australia (WA)	Retrospective	53	Maternal age– 26.9	Low	Aboriginal mothers were younger in age, and have a higher gravida than non-Indigenous mothers.
				RHD severity (AU) –severe– 40.7%		
				Previous operated– 18.5%		
Sullivan EA, et al. (2019) [42]	Australia and New Zealand	Prospective	314	Maternal age 27 (22–32)	Low	Aboriginal mothers were younger in age, more likely to smoke in pregnancy, present late to antenatal care, and be in Quintile 1(most) of social disadvantage.
				NYHA III/IV– 2.3%		
Michaelson-Cohen, et al. (2011) [30]	Israel, Jerusalem	Prospective	71	Maternal age– 32 +/-5.9	Moderate	Did not state patients who were on anticoagulants/past surgery.
				NYHA III/IV– 25.35%		
						SGA definition was <5% predicted weight
Thanajira-prapra et al. (2009) [37]	Thailand, Bangkok	Retrospective	133	Maternal age– 27.9 +/-5.8	Moderate	The most severe NYHA III/IV is recorded
				NYHA III/IV– 15%		
				Previous operated– 20%		
Rezk M, et al. (2015) [40]	Egypt, Meoufia	Prospective	192	Maternal age– 23–24 +/- 3,2	Low	Excluded co-morbidities in cohort.
				NYHA III/IV– 41.6%		
				Previous operated– 62.5%		
Pratibha D, et. al (2009) [32]	Egypt, Telangana	Retrospective	203	Maternal age– 95% between 20–30 years old	Moderate	No record of previous cardiac surgery.
				NYHA III/IV– 27.6%		
				Previous operated– 3.4%		
Silverside C et al. (2003) [35]	Canada, Toronto	Prospective	80	Maternal age– 32 +/- 5	Moderate	No clear definition of fetal outcomes.
				NYHA III/IV– 0%		Univariate analysis was not conducted for other potential confounders.
				Severe MS– 11%		
				Previous operated– 34%		

SGA/IUGR- small for gestational age/intra-uterine growth restriction; LBW-low birth weight; NYHA-New York Heart Association; MVA-mitral valve area; MS-mitral stenosis; RHD-rheumatic heart disease; NA-not applicable; GA-gestational age.

### Study characteristics and risk of bias assessment

The studies, published from 1990–2020, comprising 3928 pregnancies with rheumatic heart disease were conducted across countries including India (7), Israel (2), South Africa (2), Nepal (2), Egypt (2), Canada (1), Australia (3), Brazil (1), Thailand (1). One study involved over 60 countries using the Registry of Pregnancy and Cardiac disease (ROPAC).

**Fig 2:**
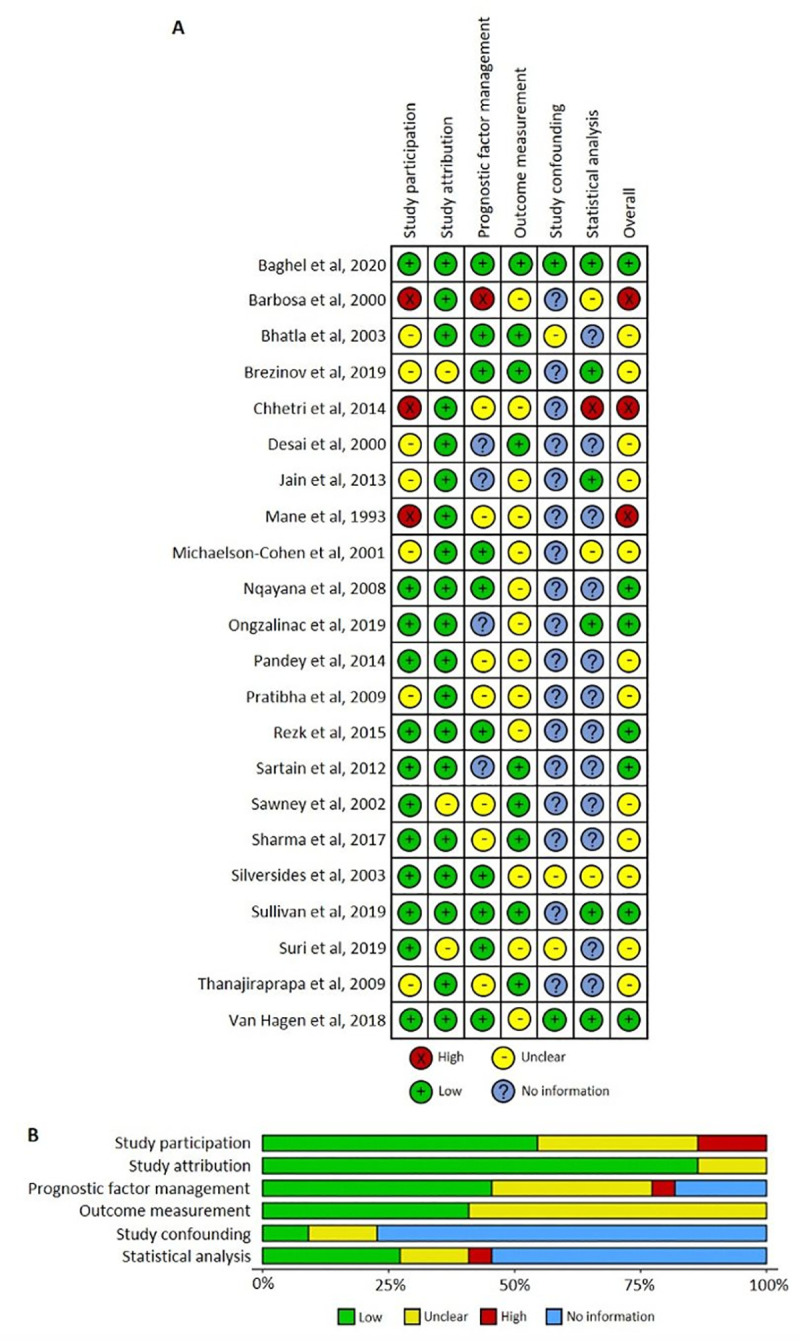
Risk of bias assessment for included studies and risk of bias summary.

Three studies were assessed as high [24–26], 12 moderate [8, 27–37], and 7 low risk [7, 9, 38–42] of bias (Fig 2). Risk of bias was frequently identified in outcome measurements, which often lacked definition, citing information bias towards the null value. Other risk of bias (3 studies) [24–26] identified were study participation. One study [24] had high risk of bias in the prognostic factor measurement (NYHA) with values being recorded post-pregnancy. Most studies were at unknown risk for confounding bias. Eight studies conducted analysis of fetal outcomes by prognostic factors using univariate analyses. Only Van Hagen et al. [9] used multivariate analysis in order to adjust for confounding. As such, results from this review regarding risk index of NYHA and MVA should be interpreted in terms of absolute risk, and meta-analysis is of unadjusted rates.

**Fig 3:**
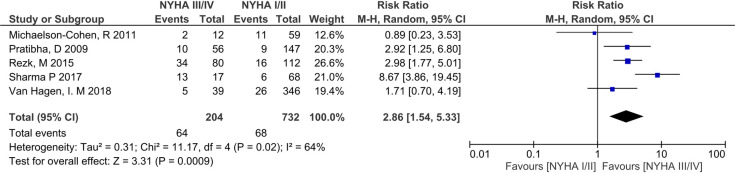
Comparison of New York health assessment I/II and New York health assessment III/IV scores for preterm births in women with baseline New York health assessment scores.

**Fig 4:**
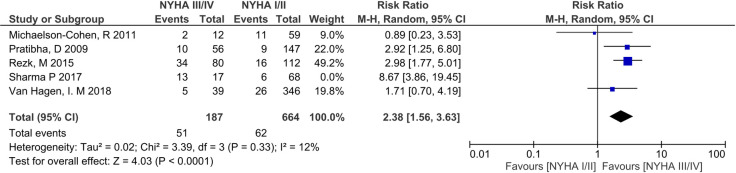
Sensitivity analyses: Comparison of New York health assessment I/II and New York health assessment III/IV scores for preterm births in women with baseline New York health assessment scores.

Preterm birth was the most commonly reported adverse outcome for women with RHD during pregnancy (Table 2) [7–9, 24–35, 37, 38, 40–42]. Incidence ranged from 9.35%-42.97%, with substantial intra- and inter-country variation; Australia [39, 41, 42], (10.81%-21.01%), India (12.00% -25.12%) [7, 26, 31, 33, 36], Nepal (15.55%-22.35%) [25, 34], Egypt (9.36%-26.04%) [32, 40] and South Africa (16.88%-41.97%) [28, 38]. Meta-analysis of preterm birth in women with baseline NYHA included 5 studies (n = 936 pregnancies) [9, 30, 32, 34, 40] and showed a clear difference in this outcome between NYHA III/IV and NYHA I/II, with a RR 2.86 (95% CI 1.54–5.33, p<0.001). Heterogeneity was high (I^2^ = 64%) (Fig 3). An outlier study from Nepal, Sharma et al [34] reported a RR of 8.67 (3.86–19.45) (Fig 3) in comparison to a RR <3 in all other studies. A post-hoc sensitivity analysis with removal of this outlier result gave an RR of 2.38 (95% CI 1.56–3.64, p<0.001), and reduced I^2^ to 12% (Fig 4).

**Table 2:** Incidence of fetal events (%).

Study (*author/Year)	n	Preterm	SGA /IUGR	LBW (<2500g)	Perinatal Death		Additional findings	Comparator (NYHA, MVA, Both) or NA
					IUD/Stillbirth	Neonatal death		
Bhatla et al. (2003) [27]	183	25.12%	22.40%	32.78%	1.10%	NA	2 congenital malformation (TOF, ASD)–none on anti-coagulants	NYHA
Suri V, et al. (2019) [36]	309	NA	25.00%	Mean BW– 2.4 +/-0.6	3.14%	1.81%	Lower GA at delivery in mild/ moderate vs severe MS (P<0.005)	NYHA
Sawhney H, et al. (2002 [33]	500	12.00%	18.20%	NA	2%	NA	10 maternal deaths– 8 in NYHA III/IV, 2 in MV replacement.	NYHA
Mane SV, et al. (1993) [26]	51	17.64%	NA	17.64%*	NA	NA	SCU admission– 13.72% (1 MAS, 6 premature)	NA
Pandey U (2014) [31]	96	12.50%	8.33%	3.12%	3.12%	NA	0 miscarriage	NA
							3 deaths due to Intraventricular haemorrhage	
Shuchi J (2013) [29]	48	25.00%	6.25%	35.40%	2.10%	NA	No difference in BW, neonatal death or preterm in those undergone PBMV vs no PBMV in pregnancy. (P>0.05)	NA
							1 congenital malformation	
Brezinov OP, et al. (2019 [8]	31	16.10%	19.35%	NA	0%	0%	Total adverse event rate was higher in severe MS when compared to moderate or mild MS (HR 3.15, 95%CI 1.04–9.52) and (HR 4.06, CI 1.14–11.19), P<0.05, respectively.	MVA
Baghel J, et al. (2020) [7]	820	20.6%	8.4%	39.7%	1.7%	2.1%	35 miscarriages (4.26%)	NA
							16.7% required admission to NICU.	
Nqayana et al. (2008 [38]	77	16.88%	NA	12.98%	6.49%	1.30%	1 congenital malformation–gastroschisis (neonatal death)	NA
Desai DK, et al. (2000) [28]	128	42.97%	16.41%	NA	6.25%	NA	38% had pulmonary oedema	NA
							Miscarriage (4.7%)– 4 miscarriage in severe MS	
Sharma P, (2017) [34]	85	22.35%	NA	NA	9.41%	12.94%	All neonatal deaths were due to premature birth complications	NYHA
Chhetri S, (2014) [25]	45	15.55%	NA	Mean BW– 2.6 +/-0.5	8.80%	15.55%	Pulmonary H was in 38% of pregnancies.	NA
							Emergency caesarean in 31% of deliveries.	
Van Hagen et al. (2019) [9]	390	9.63%	9.63%	17.89%	NA	1.00%	Miscarriage– 4.13%	Both
							Multivariable–fetal adverse outcome	
							- AF–OR 1.63 (0.20–8.90)	
							- **Severe MS**–OR 3.62 (1.45–9.05)	
							- Severe MR–OR 2.59 (0.83–8.09)	
							Anticoagulation during pregnancy–OR 0.63 (0.15–2.62)	
Barbosa PJB et al. (2000) [24]	45	22.22%	NA	22.22%	2.22%		Miscarriage– 2.22%	NA
							35% of NYHA IV had PBMV during surgery	
Sartain JB, et al. (2012) [41]	74	10.91%	NA	NA	0.00%	0.00%	9.5% with RHD did not receive antenatal care during pregnancy.	NA
Ongzalima C, et al. (2019) [39]	53	NA	NA	NA	1.83%	1.83%	Antenatal attendance was higher in non-Indigenous population than Aboriginal mothers. P = 0.0078)	NA
							Miscarriage– 1.85%	
Sullivan EA, et al. (2019) [42]	314	21.01%	NA	14.97%	2.22%	NA	Higher NICU admission, LBW in Aboriginal mothers (vs Maori and other), p<0.05	NA
							Late diagnosis of RHD was associated with low Apgar babies p<0.05	
Michaelson-Cohen, et al. (2011) [30]	71	25.35%	NA	NA	0.00%		Higher preterm deliveries in RHD (25–38%) compared to Congenital disease (13–14%, P = 0.062)	NYHA
Thanajira-prapra et al. (2009) [37]	133	11.28%	6.76%	9.77%	0.01%		1 reported birth asphyxia	NA
Rezk M, et al. (2015) [40]	192	26.04%	19.79%	NA	2.60%	3.12%	Higher NICU admission in NYHA III/IV (47.5%) vs NYHA I/II (25.8%), P<0.001	NYHA
Pratibha D, et al. (2009 [32]	203	9.36%	9.36%	37.44%	4.90%	1.00%	27.09% admitted to NICU	NYHA
Silverside C et al. (2003 [35]	80	21.25%	7.50%	NA	2.50%			MVA

SGA/IUGR- small for gestational age/intra-uterine growth restriction; LBW-low birth weight; IUD-intrauterine death; NYHA-New York Heart Association; MVA-mitral valve area; TOF-tetralogy of fallot; ASD-Atrial septal defect; GA-gestational age; SCU-special care nursery; MV-mitral valve; MR-mitral regurgitation; NICU- neonatal intensive care unit; BW-birth weight, MS-mitral stenosis; RHD = rheumatic heart disease; NA-not applicable; PBMV- percutaneous balloon mitral valvuloplasty.

**Fig 5:**
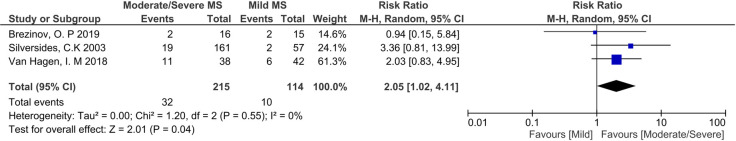
Comparison of mild and moderate/severe mitral stenosis for preterm births.

Preterm birth in women with moderate or severe MS, meta-analysis of 3 studies (n = 329) [8, 9, 35] had a significant unadjusted RR of 2.05 (95% CI 1.02–4.11, p = 0.04) (Fig 5).

**Fig 6:**
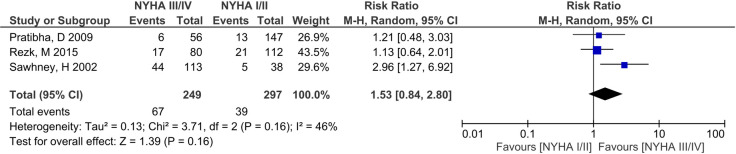
Comparison of New York health assessment I/II and New York health assessment III/IV for Intra-uterine growth restriction/small for gestational age outcome.

**Fig 7:**
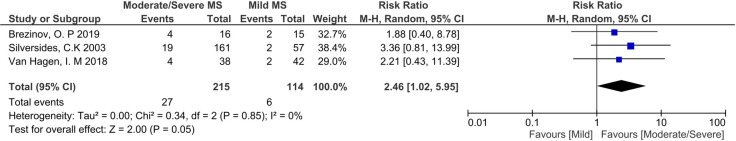
Comparison of mild and moderate/severe mitral stenosis severity for intra-uterine growth restriction/small for gestational age outcome.

Incidence of IUGR/SGA was 6.25% -25.00% among 13 studies (Table 2) [7–9, 27–39, 31–33, 35–37, 40]. On meta-analyses of 3 studies (n = 546) [32, 33, 40], NYHA III/IV was not significantly associated with IUGR/SGA (RR 1.53, 95% CI 0.84–2.80, p = 0.16) (Fig 6), but moderate/severe MS was significant (3 studies, n = 421, RR 2.46, 95% CI 1.02–5.95, p = 0.05) (Fig 7) [8, 9, 35] Subgroup analyses were not undertaken due to the limited number of studies.

**Fig 8:**
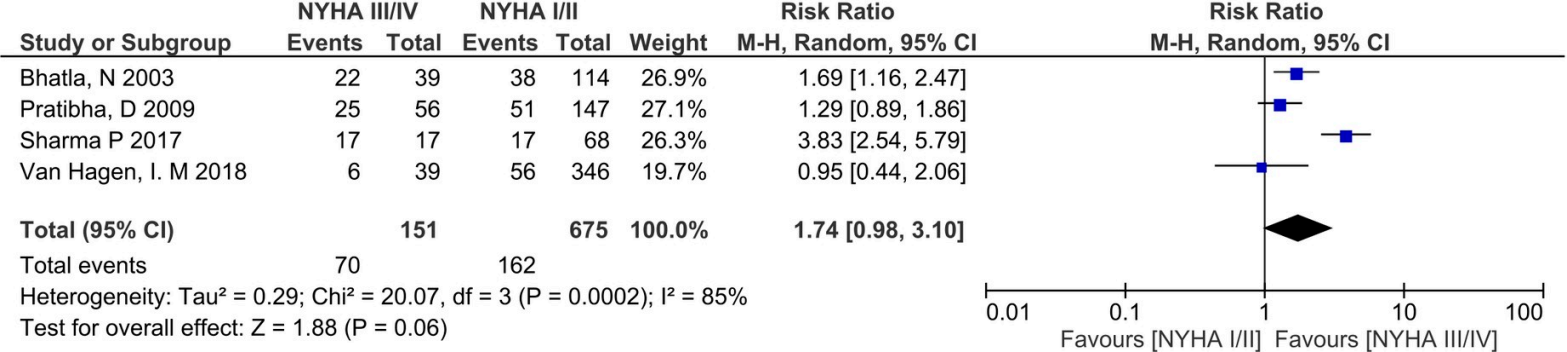
Comparison of New York health assessment I/II and New York health assessment III/IV for low birth weight outcome.

**Fig 9:**
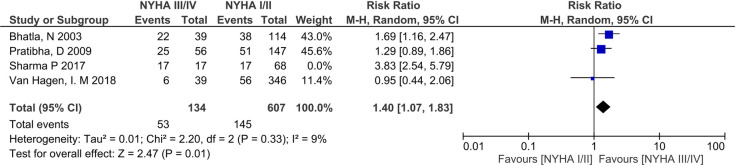
Sensitivity analyses—comparison of New York health assessment I/II and New York health assessment III/IV for low birth weight outcome.

Low-birth weight (LBW) rates varied between countries. High rates were seen in India (32.78–39.70%) [27, 29], Egypt (37.44%) [32], and Brazil (22.22%) [24], compared to Australia (14.97%) [42] and South Africa (12.98%) (Table 2) [38]. The ROPAC study^9^ reported LBW rates of 17.89% across the multiple countries included in the registry. Meta-analysis of 4 studies (n = 826) [9, 27, 32, 34] found no significant association of NYHA III/IV with LBW (RR 1.74, 95%CI 0.98–3.10, p = 0.06) and had high heterogeneity (I^2^ = 85%) (Fig 8). Post-hoc sensitivity analysis excluding the outlier study [34] changed the overall significance (RR 1.40, 95%CI 1.07–1.83, P = 0.01), and reduced statistical heterogeneity (I^2^ = 9%) (Fig 9).

Perinatal death was reported in most studies [7–9, 24–42]. Incidence of intrauterine death (IUD) (or stillbirth) varied (0.00%-9.41%), with the highest rates seen in Nepal (8.89%-9.41%) [25, 34] and South Africa (6.25%-6.49%) [28–38], as did neonatal death rates (0.63%-3.10%) (Table 2) [7, 32, 34, 38–40].

**Fig 10:**
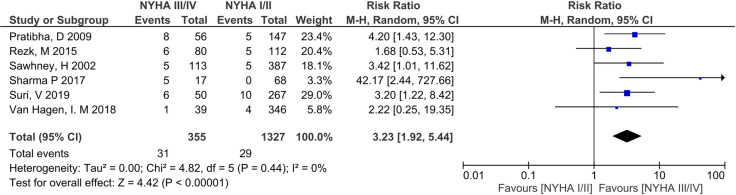
Comparison of New York health assessment I/II and New York health assessment III/IV for perinatal death outcome.

**Fig 11:**
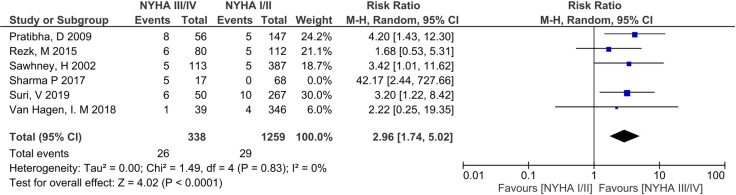
Sensitivity analyses–comparison of New York health assessment I/II and New York health assessment III/IV for perinatal death outcome.

Meta-analysis of the association of perinatal death and NYHA III/IV pre-pregnancy in 6 eligible studies (n = 1682) [9, 32–34, 36, 40] gave an unadjusted RR 3.23 (95% CI 1.92–5.44, p<0.001). Heterogeneity was low (I^2^ = 0%) (Fig 10). Sharma et al. [28] was a clear outlier with a neonatal mortality rate of 12.94%. Post-hoc sensitivity analysis after exclusion of this study gave an RR of 2.96 (1.74–5.02, p<0.001) (Fig 11).

Intervention with percutaneous balloon mitral valvuloplasty (PBMV) during pregnancy compared to no intervention was reported in 3 studies [29, 32, 36]. Suri et al. reported lower birth GA [36], and higher perinatal deaths in those with NYHA III/IV who did not undergo PBMV vs those who did; (GA 37.15+/-1.06 vs 43.8+/-3.61, p = 0.002), and (0% perinatal death vs 19.4%, p = 0.08) respectively [36]. Lower preterm births in women who underwent PBMV during pregnancy was also seen in another study [29] but did not reach statistical significance. No meta-analysis was conducted for this outcome.

Incidence of miscarriage in pregnancy with RHD was investigated in 5 studies [7, 24, 28, 31, 42] Rates varied between 1.85%^39^–4.70% [28]. One study found 4 out of the 6 miscarriages in their cohort were attributed to critical MS <1.0cm^2^ [28]. No studies had a comparator group for meta-analysis. Congenital malformations were rarely reported [27, 29, 38].

High Neonatal Intensive Care Unit (NICU) admission rates (13.70–42.25%) were reported in 3 studies [7, 26, 32]. One study found significantly higher NICU admissions associated with NYHA III/IV pre-pregnancy in women with RHD vs NYHA I/II (42.50% vs 14.20%, P<0.001) [40].

RHD was first diagnosed during pregnancy in 66.5% of patients in one study [32]. High rates were also seen in Australia (14.2%) [42], South Africa (42%) [28] and 24.9% in the ROPAC study [9]. Limited antenatal care in multiple studies [25, 34, 39, 42] was associated with poor fetal outcomes and late optimisation of anticoagulants during pregnancy in select women.

One study [9] conducted adjusted analysis and found severe MS was independently associated with adverse fetal outcomes (OR 3.62, 95%CI 1.45–9.05), when adjusted for atrial fibrillation, severe mitral regurgitation, and anticoagulation during pregnancy. Pre-pregnancy NYHA>1 did not show univariate significance with adverse fetal outcomes (OR 1.10, 95% CI 0.59–2.02, p = 0.10), but was an independent predictor of maternal cardiac events in women with MS [9].

Funnel plot and Eggers test was not conducted as less than 10 studies were included per meta-analysis. The GRADE system rated the overall certainty of evidence as low for MVA and NYHA as markers for preterm, and very low for NYHA as markers of SGA/IUGR, LBW and perinatal death (Table 3). There was also low certainty for MVA and SGA/IUGR.

**Table 3:** Assessment of quality of evidence of NYHA and MVA as risk indexes for adverse fetal events in RHD during pregnancy by the Grades of Recommendation Assessment, Development and Evaluation (GRADE) approach.

	No. studies	Design	Risk of bias	Inconsistency	Indirectness	Imprecision	Certainty (overall score)*
**Preterm**							
NYHA	5	Observational	Low	Low^A^	Low	Low	Low⊕⊕◯◯
MVA	3	Observational	Low	Low	Low	Low	Low⊕⊕◯◯
**SGA/IUGR**							
NYHA	3	Observational	Low	Low	Low	Very Low^B^	Very Low⊕◯◯◯
MVA	3	Observational	Low	Low	Low	Low	Low⊕⊕◯◯
**LBW**							
NYHA	4	Observational	Low	Low	Low	Very Low^C^	Very Low⊕◯◯◯
MVA	NA						
**Perinatal Death**							
NYHA	6	Observational	Low	Low	Very low^D^	Low	Very Low⊕◯◯◯
MVA	NA						

* Meta-analysis of observational studies has an initial confidence estimate of ‘Low confidence’[43]. No outcome was rated up in confidence as analysis did not yield a sufficiently large effect size.

A- The evidence was kept as low after sensitivity analyses (95%CI 1.56–3.64, I^2^ = 12%) removing an outlier study.

B- The evidence was downgraded to very low due to the significant difference in clinical implications of the lower and upper limits of the overall CI (0.84–2.80).

C- The evidence was downgraded as the CI is wide and crosses no significant effect CI (0.98–3.10).

D- The evidence was downgraded to very low as perinatal death did not specify between women with PBMV vs non-PBMV, or women on anticoagulant. Both factors have shown to significantly influence maternal mortality (and fetal death) [44, 45].

## Discussion

Evidence from the 22 included studies suggest RHD in pregnancy is associated with high rates of adverse fetal outcomes (preterm birth, LBW, SGA, IUGR, miscarriage and perinatal death). Additional outcomes found high rates of NICU admissions [26, 32, 40] low rates of antenatal care and late diagnosis of RHD in many women.

On meta-analysis, both severe MS and NYHA III/IV were significantly associated with preterm birth. Additionally, NYHA III/IV were also associated with higher rates of perinatal death. A Nepalese study [34] appeared to be an outlier in reporting consistently higher rates of fetal adverse outcomes in their patient cohort. Post-hoc sensitivity analyses excluding this study lowered statistical heterogeneity and reduced the RR. This could indicate that this site in Nepal may have wider health care inequities (low health resources, health access, co-morbidities) compared to other developing countries (India, Egypt etc.).

The association between rheumatic MS during pregnancy and adverse fetal outcomes is biologically plausible. Early pregnancy is associated with a 30–40% increase in cardiac preload [43], decreased systematic vascular resistance and systolic blood pressure. These changes are poorly tolerated in MS and restricted left ventricular inflow with increasing atrial pulmonary pressures often precipitates cardiac decompensation and pulmonary edema [10]. Adverse fetal outcomes are likely due to uteroplacental insufficiency secondary to left heart obstruction [10]. Poor oxygen and nutrient transfer may lead to stunted fetal growth.

Mitral stenosis carries a high risk of chronic fetal hypoxia and early onset (<32 weeks) IUGR in pregnancy. These fetuses are more likely born preterm, and are high risk of rapid deterioration, fetal demise in-utero and stillbirth [44]. There are also recognised links of IUGR with cardio-vascular remodelling, sub-optimal renal and neurological development, and altered glucose metabolism; collectively known as the fetal origin hypothesis [45]. Such outcomes are currently unexplored in neonates born to mothers with RHD.

The prognostic value of NYHA classification for neonatal outcomes is likely a reflection of the severity of the pressure gradient across the mitral valve and underlying pulmonary edema. As such, it is well established in predicting maternal cardiac events, but less so for adverse fetal events. This review found NYHA class III/IV had significant associations with prematurity and perinatal death, but not LBW or IUGR/SGA. Conversely, mitral valve area (MVA) determined by echocardiogram could more directly indicate cardiac output and uteroplacental perfusion as moderate/severe MS was significantly associated with both SGA/IUGR and prematurity.

RHD remains the predominant form of maternal heart disease in pregnancy in developing nations [3, 4]. In this study, developing countries [46] (India, Nepal, Egypt, South Africa) exhibited relatively higher rates of adverse neonatal outcomes compared to developed countries (Australia, New Zealand). Shortage of health services and delayed access to tertiary centres may be more evident in these developing nations, with further limited capacity of hospitals in surgical intervention [29] and neonatal intensive care [34].

Poorer education among women with RHD was reported in one study in Chandigarh, India [36]; 14% illiterate and 10.6% only receiving a primary school education. Downstream health behaviours associated with low education status, such as younger maternal age and multiparity are also predictors for adverse perinatal events [47].

Within-country variations in birth outcomes were observed in western developed nations. One study found higher rates of preterm and perinatal death in Aboriginal Australians or Torres Strait Islanders, and Maori or Pasifika mothers compared to non-Indigenous counterparts [42]. Indigenous mothers with RHD were significantly younger, [39, 42] more likely to present >20 weeks to antenatal clinic, be socioeconomically disadvantaged, and smoke during pregnancy compared to non-Indigenous mothers [42]. While the disparity in fetal outcomes is likely a combination of these bio-psychosocial factors, there is evidence of an independent association of RHD in pregnancy. For example, in Australia, one study [42] reported an overall preterm birth rate of 21%; much higher than the overall rate of Australia (9%) and of babies born to Indigenous mothers (14%) [48] As such, closing the gap between health inequities among disadvantaged populations is a priority in the improvement of global neonatal health, and eradication of RHD among women of child-bearing age.

Antenatal care remains a critical component of neonatal outcomes in RHD patients [49]. Sub-optimal antenatal visits were common among studies with relatively higher adverse fetal events. In one study [25] over 90% of women presented for first time in labour and reported a high IUD rate (6.25%). In Durban (South Africa) [38] 62% had first cardiac evaluation in 3^rd^ trimester and had 42.97% prematurity deliveries. Greater emphasis on pregnancy planning, particularly after an index pregnancy would be beneficial.

Pregnancy planning and early initial antenatal consultation is also important for women with a surgical valve replacement and on lifelong anticoagulants such as warfarin. Delayed initial antenatal visits and low uptake of contraceptives [50] were reported among this sub-group of women in several studies [34, 38]. This is concerning as warfarin is teratogenic and has strong associations with fetal malformation, abortion and stillbirth. Early optimisation with heparin or low-molecular weight heparin should be a priority in these patients [51].

PBMV remains the treatment of choice for isolated non-calcified MS and is safe to perform during pregnancy, with few adverse maternal or fetal events [52–54]. This systematic review suggests PBMV in women with severe symptoms (NYHA III/IV) is associated with reduced rates of preterm births; however further comparative studies are required. No long-term effects on child development have been reported to date [52, 55, 56].

Mitral valve surgery involving cardiac bypass was not assessed in this review as such interventions are avoided where possible during pregnancy due to the significantly high associated fetal mortality (ranging from 5–33%) [54, 57].

## Limitations

Neonatal outcomes are influenced by a complex interplay of known and unknown factors. RHD is associated with socio-economically disadvantage, and many important potentially confounding variables such as smoking, poor antenatal care, chronic disease [58] and extent of RHD-related antenatal services in hospitals of different countries, which were not measured in the included studies. Insufficient reporting on outcomes of women on warfarin during pregnancy also limited analysis of a clinically important sub-group.

Only studies based at tertiary hospitals were included, and it is likely that lower-resourced and rural areas experience even poorer pregnancy outcomes that are underreported.

There is some methodological limitation in this research. First, most studies in this review had moderate risk of bias in multiple domains. In particular, outcome definitions were not clearly explained, and influence of confounders was also uncertain. Second, there is a possibility of publication bias among studies with small samples, particularly in outcomes around perinatal death. Overestimation of the true effect size may have resulted from smaller studies with non-significant findings not being published. Third, there was clinical and statistical heterogeneity between studies. The overall certainty of the evidence generated from meta-analysis was low or very low (Table 3), although the GRADE system allows a maximum of low-quality evidence for meta-analysis of cohort studies [59].

These findings are important from a national, international, public health policy perspective, highlighting increased perinatal morbidity and mortality in infants born to women with RHD. As our results indicate that moderate or severe MS, symptomatic NYHA, have worse outcomes, we recommend early specialist involvement in these cases. While no definitive management for IUGR exists besides delivery, neonatal USS Doppler could be useful for early identification. PBMV in pregnancy is an effective, low risk procedure for symptom relief in MS during pregnancy but requires further research. Finally, our findings add support to large scale echocardiographic screening of RHD in pregnancy in high-risk populations.

Large, well-designed prospective studies of pregnancy in women with RHD are required. Associations with NYHA and MVA severity on neonatal outcomes need to be calculated based on adjusted rates. One study [9] in this systematic review demonstrated robust methodology that could be modelled in future studies.

## Supporting information

S1 Checklist PRISMA 2009 checklist. (DOC)

S1 Fig Literature search strategy. (DOCX)

S2 Fig Risk of bias assessment tool: Modified (QUIPS) template. (DOCX)
