# Supplementary material for: Rheumatic heart disease in pregnancy and neonatal outcomes: A systematic review and meta-analysis
Source: PLoS One. 2021 Jun 29;16(6):e0253581. doi: 10.1371/journal.pone.0253581 (PMC8241043; doi:10.1371/journal.pone.0253581)
Supplement: S1 Fig — (DOCX) [file pone.0253581.s002.docx]

**Supplemental 1 – Literature search strategy**

The search strategy is divided into 3 groups of keywords

| Population | #1 Women in Pregnancy |
| --- | --- |
| Intervention | #2 Rheumatic Heart Disease or Mitral stenosis |
| Outcomes of Interest | #3 Perinatal and Pregnancy outcomes |

The three groups were combined with the following logic (**#1 OR #3) AND #2**

#### MEDLINE (OvidSP) n=886

("gestation"[All Fields] OR "pregnancies"[All Fields] OR "pregnancy"[All Fields] OR

"pregnancy complication"[All Fields] OR "pregnancy complications"[All Fields] OR "postnatal period"[All Fields] OR "postnatal periods"[All Fields] OR "obstetric labor complications"[All Fields] OR "obstetric"[All Fields] OR "obstetric emergencies"[All Fields] OR "Pregnancy outcome"[All Fields] OR "pregnancy outcomes"[All Fields] OR "Maternal health care"[All Fields] OR "maternity care"[All Fields] OR "maternal-child health"[All Fields] OR "Maternal mortality"[All Fields] OR "maternal mortalities"[All Fields] OR "Maternal death"[All Fields] OR "maternal deaths"[All Fields] OR "cardiovascular pregnancy complication"[All Fields] OR "cardiovascular pregnancy complications"[All Fields] OR "Fetal death"[All Fields] OR "fetal deaths"[All Fields] OR "fetal demise"[All Fields] OR "Maternal morbidity"[All Fields] OR "fetal morbidity"[All Fields] OR "gestational hypertension"[All Fields] OR "pregnancy induced hypertension"[All Fields] OR "neonatal"[All Fields] OR "prematurity"[All Fields] OR "neonate"[All Fields]) AND

("rheumatic fever"[All Fields] OR "rheumatic fevers"[All Fields] OR "acute rheumatic arthritis"[All Fields] OR "acute rheumatic fever"[All Fields] OR ("rheumatic fever"[MeSH Terms] OR ("rheumatic"[All Fields] AND "fever"[All Fields]) OR "rheumatic fever"[All Fields] OR ("acute"[All Fields] AND "rheumatic"[All Fields] AND "fevers"[All Fields])) OR "Rheumatoid fever"[All Fields] OR "rheumatic fever prevention"[All Fields] OR "bouillaud disease"[All Fields] OR "bouillaud's disease"[All Fields] OR ("rheumatic heart disease"[MeSH Terms] OR ("rheumatic"[All Fields] AND "heart"[All Fields] AND "disease"[All Fields]) OR "rheumatic heart disease"[All Fields] OR ("bouillauds"[All Fields] AND "disease"[All Fields])) OR "rheumatic heart disease"[All Fields] OR "rheumatic heart diseases"[All Fields] OR "mitral valve"[All Fields] OR "mitral valves"[All Fields] OR "mitral stenosis"[All Fields] OR "mitral valvuloplasty"[All Fields] OR "mitral valve annuloplasty"[All Fields] OR "mitral replacement"[All Fields] OR "mitral valve insufficiency"[All Fields] OR "mitral valve diseases"[All Fields])

#### CINAHL n=1650

(MH "Pregnancy+") OR (MH "Pregnancy, Multiple+") OR (MH "Pregnancy Trimesters+") OR (MH "Postnatal Period+") OR (MH "Maternal-Child Health") OR (MH "Maternal Health Services") OR (MH "Indigenous Health")

(MH "Mitral Valve") OR (MH "Mitral Valve Insufficiency") OR (MH "Mitral Valve Annuloplasty") OR (MH "Mitral Valve Stenosis") OR (MH "Mitral Valve Diseases+") OR (MH "Mitral Valve Prolapse") OR (MH "Rheumatic Heart Disease") OR (MH "Rheumatic Fever+") 

(MH "Obstetric Emergencies")OR(MH "Pregnancy Complications+") OR (MH "Pregnancy Outcomes") OR (MH "Outcomes of Prematurity") OR (MH "Pregnancy, Ectopic") OR (MH "Treatment Outcomes+") OR

#### INFORMIT n=216 results

("rheumatic fever" OR "rheumatic fevers" OR "acute articular rheumatism" OR "acute rheumatic arthritis" OR "acute rheumatic fever" OR "acute rheumatic fevers" OR "Rheumatoid fever" OR "rheumatic fever prevention" OR ""bouillaud disease" OR "bouillaud's disease" OR "bouillauds disease" OR "rheumatic heart disease" OR "rheumatic heart diseases" OR ""bicuspid valve" OR "mitral valve" OR "mitral valves" OR "cardiac valve" OR "cardiac valves" OR "heart valve" OR "heart valves" OR "mitral stenosis" OR "mitral valvuloplasty" OR "mitral valve annuloplasty" "mitral replacement" or "valvular heart disease" OR "mitral valve insufficiency" OR "mitral valve diseases") AND

("gestation" OR "pregnancies" OR "pregnancy") OR

("pregnancy complication" OR "pregnancy complications" OR "postnatal period" OR "postnatal periods" OR obstetric labor complications" OR "obstetric" OR "obstetric emergencies" OR "Treatment Outcomes" OR ""Pregnancy outcome" OR "pregnancy outcomes" OR ""Maternal health care" OR "maternity care" OR "maternal-child health" OR "Maternal Health services" OR "indigenous health" OR "Maternal mortality" OR "maternal mortalities" OR "Maternal death" OR "maternal deaths" OR ""cardiovascular pregnancy complication" OR "cardiovascular pregnancy complications" "infectious pregnancy complication" OR "infectious pregnancy complications" OR "maternal sepsis" OR "sepsis during pregnancy" OR "sepsis in pregnancies" OR "sepsis in pregnancy" OR "sepsis during pregnancies" OR "sepsis during pregnancy" OR ""Fetal death" OR "fetal deaths" OR "fetal demise" OR "fetal mummification" OR ""Maternal morbidity" OR "fetal morbidity" OR "gestational hypertension" OR "pregnancy induced hypertension" OR "neonatal" OR "prematurity" OR "neonate" OR "infant")

#### MEDLINE (PUBMED) n=2893

("gestation"[All Fields] OR "pregnancies"[All Fields] OR "pregnancy"[All Fields] OR "pregnancy complication"[All Fields] OR

"pregnancy complications"[All Fields] OR "postnatal period"[All Fields] OR "postnatal periods"[All Fields] OR "obstetric labor complications"[All Fields] OR "obstetric"[All Fields] OR "obstetric emergencies"[All Fields] OR "Pregnancy outcome"[All Fields] OR "pregnancy outcomes"[All Fields] OR "Maternal health care"[All Fields] OR "maternity care"[All Fields] OR "maternal-child health"[All Fields] OR "Maternal mortality"[All Fields] OR "maternal mortalities"[All Fields] OR "Maternal death"[All Fields] OR "maternal deaths"[All Fields] OR "cardiovascular pregnancy complication"[All Fields] OR "cardiovascular pregnancy complications"[All Fields] OR "Fetal death"[All Fields] OR "fetal deaths"[All Fields] OR "fetal demise"[All Fields] OR "Maternal morbidity"[All Fields] OR "fetal morbidity"[All Fields] OR "gestational hypertension"[All Fields] OR "pregnancy induced hypertension"[All Fields] OR "neonatal"[All Fields] OR "prematurity"[All Fields] OR "neonate"[All Fields]) AND

("rheumatic fever"[All Fields] OR "rheumatic fevers"[All Fields] OR "acute rheumatic arthritis"[All Fields] OR "acute rheumatic fever"[All Fields] OR ("rheumatic fever"[MeSH Terms] OR ("rheumatic"[All Fields] AND "fever"[All Fields]) OR "rheumatic fever"[All Fields] OR ("acute"[All Fields] AND "rheumatic"[All Fields] AND "fevers"[All Fields])) OR "Rheumatoid fever"[All Fields] OR "rheumatic fever prevention"[All Fields] OR "bouillaud disease"[All Fields] OR "bouillaud's disease"[All Fields] OR ("rheumatic heart disease"[MeSH Terms] OR ("rheumatic"[All Fields] AND "heart"[All Fields] AND "disease"[All Fields]) OR "rheumatic heart disease"[All Fields] OR ("bouillauds"[All Fields] AND "disease"[All Fields])) OR "rheumatic heart disease"[All Fields] OR "rheumatic heart diseases"[All Fields] OR "mitral valve"[All Fields] OR "mitral valves"[All Fields] OR "mitral stenosis"[All Fields] OR "mitral valvuloplasty"[All Fields] OR "mitral valve annuloplasty"[All Fields] OR "mitral replacement"[All Fields] OR "mitral valve insufficiency"[All Fields] OR "mitral valve diseases"[All Fields])

#### SCOPUS n=3863

( ( TITLE-ABS-KEY ( "rheumatic heart disease" OR "rheumatic heart diseases" OR "bicuspid valve" OR "mitral valve" OR "mitral valves" ) ) OR ( TITLE-ABS-KEY ( "rheumatic fever" OR "rheumatic fevers" OR "acute rheumatic fever" OR "acute rheumatic fevers" OR "Rheumatoid fever" ) ) OR ( TITLE-ABS-KEY ( "mitral stenosis" OR "mitral valvuloplasty" OR "mitral valve annuloplasty" OR "mitral replacement" OR "mitral valve insufficiency" OR "mitral valve diseases" ) ) ) AND

( ( TITLE-ABS-KEY ( "Maternal morbidity" ) ) OR ( TITLE-ABS-KEY ( "Maternal death" OR "maternal deaths" OR "cardiovascular pregnancy complication" OR "cardiovascular pregnancy complications" ) ) OR ( TITLE-ABS-KEY ( "Pregnancy outcome" OR "pregnancy outcomes" OR "maternal-child health" OR "Maternal mortality" OR "maternal mortalities" ) ) OR ( TITLE-ABS-KEY ( "gestation" OR "pregnancies" OR "pregnancy" OR "pregnancy complication" OR "pregnancy complications" OR "postnatal period" OR "postnatal periods" OR "obstetric labor complications" OR "obstetric emergencies" OR "Perinatal" ) ) )

#### ICTRP n=57

"rheumatic fever" OR "rheumatic heart disease" OR "Acute rheumatic fever"

#### EMCARE n=154

exp rheumatic fever/ or exp rheumatic heart disease/exp Mitral Valve/

Pregnancy Outcome/ or exp pregnancy complications/ or exp fetal death/ or exp hypertension, pregnancy-induced/ or maternal death/ or exp obstetric labor complications/ or exp pregnancy complications, cardiovascular/ or exp pregnancy complications, hematologic/ or exp pregnancy complications, infectious/ or exp pregnancy complications, neoplastic/ or exp pregnancy, ectopic/ or pregnancy, prolonged/

maternal care/ or maternal death/ or maternal child health care/ or maternal health service/ or maternal morbidity/ or maternal mortality/
