# Supplementary material for: Rheumatic heart disease in pregnancy and neonatal outcomes: A systematic review and meta-analysis
Source: PLoS One. 2021 Jun 29;16(6):e0253581. doi: 10.1371/journal.pone.0253581 (PMC8241043; doi:10.1371/journal.pone.0253581)
Supplement: S2 Fig — (DOCX) [file pone.0253581.s003.docx]

### **Supplemental 2 – Risk of Bias Assessment Tool: Modified (QUIPS) template**

| **Component** | **Aspects** | **Yes/No/Unclear** | **Justification** | **High Risk** | **Moderate Risk** | **Low Risk** |
| --- | --- | --- | --- | --- | --- | --- |
| **Study participation** | Source population of interest – Country and Tertiary hospital. |  |  | Study represents a very limited population (patients with RHD in pregnancy) AND/OR has a limited sample size  AND/OR study does not report adequate  characteristics of the population  Highly likely to have bias of observed outcomes for participants and eligible non-participants. | Sample size may be limited  OR  modestly represents a population of patients with RHD in pregnancy.  AND/OR  Baseline characteristics are mostly accommodated.  May be biased in observed outcomes +/- relationship with PF. | Sample size is sufficient, AND adequately represents a population of patients with RHD in pregnancy.  AND  Baseline Characteristics are sufficiently reported.  Very unlikely bias of observed outcome +/- relationship with PF |
|  | Adequate description of participants recruitment (Time frame, method of RHD diagnosis) |  |  |  |  |  |
|  | Adequate description of inclusion and exclusion  (logical and unlikely to bias observed outcomes) |  |  |  |  |  |
|  | Adequate participation of the study by eligible participants – (n >50 pregnancies) |  |  |  |  |  |
|  | Description of baseline study adequate in key characteristics?   - Yes = ALL (-1) of Major, most of Minor - No = <half of Major, AND/OR <2 Minor |  |  |  |  |  |
|  | MAJOR | |  |  |  |  |
|  | NYHA status |  |  |  |  |  |
|  | Age |  |  |  |  |  |
|  | Mode of Delivery |  |  |  |  |  |
|  | RHD specific lesions |  |  |  |  |  |
|  | Previous Cardiac Surgery |  |  |  |  |  |
|  | On Medications – anticoagulation AND cardiac medications |  |  |  |  |  |
|  | MINOR | |  |  |  |  |
|  | Parity/Gravida |  |  |  |  |  |
|  | Co morbidities |  |  |  |  |  |
|  | Antenatal care (and time of first admission) |  |  |  |  |  |
|  | BMI |  |  |  |  |  |
|  | MVA of mitral valve |  |  |  |  |  |
|  | Mean Gestational Age |  |  |  |  |  |
|  | Ethnicity (if applicable) |  |  |  |  |  |
| **Study Attribution** | Response rate is >80% of total sample size |  | ** can be NA (= low risk bias for study attribution)* | The relationship between the sample key characteristics and  outcome is very likely to be  different for completing and  noncompeting participants | The relationship between the sample key characteristics and outcome is unclear OR may be different, for completing and non-completing participations. | The relationship between the sample and outcome is unlikely to be different  for completing and noncompeting  participants |
|  | Reasons for loss to follow up is described adequately |  |  |  |  |  |
|  | Adequate description of participants lost/differences between participants who completed and drop out |  |  |  |  |  |
| **Prognostic Factor Measurement (NYHA)** | Clear definition of prognostic factor was made. |  |  | Measurement of NYHA class was incorrect, and/or very likely not consistent among participants. | Measurement of NYHA is by a reliable method, but unclear, or some discrepancy in consistency of application between participants. | Measurement of NYHA class is by a reliable, and consistent method, to sufficiently limit bias in participants. |
|  | Method of NYHA measurement is adequately valid and reliable *(see reference below)* |  |  |  |  |  |
|  | Method and setting of measurement was same for all study participants |  |  |  |  |  |
|  | >80% of study sample had completed data for NYHA. |  |  |  |  |  |
|  | Appropriate justification of missing ‘PF’ data. |  |  |  |  |  |
| **Outcome measurement**  **Primary Outcome - Pre** | **Primary Outcome – Fetal/Neonate Outcomes** | | | | | |
|  | Clear definition of the outcome of interest is provided (Neonatal/fetal outcome) |  | **record if definition different to study proposed definition.* | Outcome of interest is very inconsistent in measurement, method is not reliable and/or significant variance in setting of outcome measurement between participants  Highly likely to be biased among sample +/- relationship with PF. | Outcome of interest has some inconsistency in measurement OR has a unique definition not widely practiced.  May have potential bias in the sample +/- relationship with PF. | Outcome of interest is adequately measured AND defined in study participants to limit potential bias in the sample +/- relationship with PF. |
|  | Is the method of measurement valid and reliable and limit misclassification bias (refer to below for definition) |  |  |  |  |  |
|  | Method and setting of outcome measurement is the same for all study participants (in pregnancy, tertiary centre etc.) |  |  |  |  |  |
| **Study confounding**  **X each outcome** | *NOT relevant when the study is being reviewed for purpose of identifying ABSOLUTE risk of the outcome in the group w/ prognostic factors   - So, if they do not look at **relative** risk of NYHA classification 🡪 section not relevant) | | | | | |
|  | All-important confounders are measured with clear definitions provided (confounder = Major of Criteria 1) |  |  | Limited, or no recognition of important confounders on the observed effects of the sample population (and/or prognostic factor) on outcomes.  *Highly likely bias of relationship of RHD (and/or PF) with fetal outcome* | Some important potential confounders are appropriately accounted for,  *May have bias of relationship of RHD (and/or PF) with fetal outcome* | MOST/ALL Important confounders are appropriately accounted for.  *Very unlikely bias of relationship of RHD (and/or PF) with fetal outcome.* |
|  | Measurement of reported characteristics was accurate, valid and reliable. |  |  |  |  |  |
|  | All-important potential confounders are accounted for in the analysis (where significant). |  |  |  |  |  |
| **Statistical analysis** | Sufficient presentation of primary outcomes to adequately assess method of analysis. |  | **NA if not conducted* | Statistical analysis if completely inappropriate, and reported results are very likely spurious or biased. | Statistical analysis may be appropriate for design and may have some potential invalidities of results. | Statistical analysis is appropriate for design of study, limiting potential of invalid or spurious results |
|  | Statistical methods are appropriate/justified for study design and type of data |  |  |  |  |  |
|  | Completeness of results presented in multi-variable model – no obvious selective reporting. |  |  |  |  |  |
| OVERALL rating | | | | | | |

**(Secondary outcomes) definitions**

- Preterm defined as live delivery before 37 weeks of gestation
- LBW – composite of BW <2500g OR SGA OR IUGR (defined as BW <10%)
- Perinatal death – defined as fetal death in utero or after 20 weeks
- Miscarriage /spontaneous abortion – non-viable product of conception <20 weeks
- Long term outcomes – at min. 1 year

**Prognostic factor – NYHA classification should be made on the patient’s first admission into the hospital.**

- *(record if study reports NYHA that was measured in a different way.*
